# Complicated ventricular arrhythmia and hematologic myeloproliferative disorder in *RIT1‐*associated Noonan syndrome: Expanding the phenotype and review of the literature

**DOI:** 10.1002/mgg3.1253

**Published:** 2020-05-12

**Authors:** Safwat A. Aly, Kenneth M. Boyer, Brie‐Ann A. Muller, Davide Marini, Carolyn H. Jones, Hoang H. Nguyen

**Affiliations:** ^1^ Department of Pediatrics Rush University Medical College Chicago IL USA; ^2^ Division of Cardiology Department of Pediatrics The Hospital for Sick Children University of Toronto Toronto Ontario Canada

**Keywords:** accelerated idioventricular rhythm, monocytosis, myeloproliferative disorder, Noonan syndrome

## Abstract

**Background:**

Noonan syndrome is an autosomal dominant disorder secondary to RASopathies, which are caused by germ‐line mutations in genes encoding components of the RAS mitogen‐activated protein kinase pathway. RIT1 (OMIM *609591) was recently reported as a disease gene for Noonan syndrome.

**Methods and Results:**

We present a patient with *RIT1‐*associated Noonan syndrome, who in addition to the congenital heart defect, had monocytosis, myeloproliferative disorder, and accelerated idioventricular rhythm that was associated with severe hemodynamic instability. Noonan syndrome was suspected given the severe pulmonary stenosis, persistent monocytosis, and “left‐shifted” complete blood counts without any evidence of an infectious process. Genetic testing revealed that the patient had a heterozygous c.221 C>G (pAla74Gly) mutation in the *RIT1*.

**Conclusion:**

We report a case of neonatal Noonan syndrome associated with *RIT1* mutation. The clinical suspicion for Noonan syndrome was based only on the congenital heart defect, persistent monocytosis, and myeloproliferative process as the child lacked all other hallmarks characteristics of Noonan syndrome. However, the patient had an unusually malignant ventricular dysrhythmia that lead to his demise. The case highlights the fact that despite its heterogeneous presentation, *RIT1*‐associated Noonan syndrome can be extremely severe with poor outcome.

## Graphical abstract

RIT1 is one of the most recent genes identified in association with Noonan Syndrome. In this report, we present a patient with RIT1‐associated Noonan syndrome, who had pulmonary valve stenosis, monocytosis, myeloproliferative disorder, and hemodynamically significant ventricular dysrhythmias. Genetic testing revealed that the patient had a heterozygous c.221 C>G (pAla57Gly) mutation in the RIT1 gene.

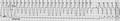

## 1 INTRODUCTION

Noonan syndrome, a common autosomal dominant disorder characterized by distinct cranio‐facial features (tall forehead, hypertelorism, down‐slanting palpebral fissures, low‐set and posteriorly angulated ears, and short webbed neck), developmental delay, short stature, cardiac abnormalities (hypertrophic cardiomyopathy, pulmonary valve stenosis, multifocal atrial tachycardia, and septal defects), skeletal abnormalities (pectus excavatum, scoliosis, and vertebral anomalies), cryptorchidism, and blood disorders (Noonan, 1968; Romano et al., 2010). There are many genes identified in association with this clinical disorder including *PTPN11* (OMIM #176876), *SOS1* (OMIM *182,530), *SOS2* (OMIM #601247), *BRAF* (OMIM *164,757), *RAF1* (OMIM *164760), *KRAS* (OMIM *190070), *NRAS* (OMIM *164790), *RRAS* (OMIM *165090), *MAP2K1* (OMIM *176872), *LZTR1* (OMIM *600574), and *RIT1* (OMIM *609591) (Aoki et al., 2013; Roberts, Allanson, Tartaglia, & Gelb, 2013; Roberts et al., 2007; Tartaglia et al., 2001). We present a patient with *RIT1* ***‐***associated Noonan syndrome, who in addition to the congenital heart defect, had monocytosis, myeloproliferative disorder, and hemodynamically significant ventricular dysrhythmias that led to his demise. This case details a novel cardiac and hematologic phenotype and illustrates the severity of *RIT1* ***‐***associated Noonan syndrome.

## 2 CASE PRESENTATION

A term baby boy, born to unrelated and healthy parents, was transferred to our hospital for management of pulmonary valve stenosis. The pregnancy was complicated by increased nuchal translucency and suspected pulmonary valve stenosis on fetal echocardiogram at 24 weeks of estimated gestation. Abnormal fetal heart rhythm was not suspected nor observed during pregnancy. There was no family history of congenital heart disease. The delivery was via spontaneous vaginal delivery at 38 weeks of estimated gestation. Birthweight was 4 kg. APGAR scores were 8 and 9 at 1 and 5 min, respectively.

Physical exam did not reveal any characteristic findings of known genetic syndromes. The postnatal echocardiogram showed a hypoplastic and thickened pulmonary valve, moderate to severe pulmonary valve stenosis, and normal biventricular systolic function. There was no evidence of obstructive hypertrophic cardiomyopathy on echocardiogram or cardiac catheterization.

On day of life 4, the patient underwent balloon pulmonary valvuloplasty to alleviate the stenosis with good result. However, worsening pressure gradient across the pulmonary valve and decreased right ventricular systolic function was seen on echocardiograms over the next 2 days. The patient underwent surgical pulmonary valvectomy and supra‐annular main pulmonary arterioplasty. The surgery was successful without complications.

**Figure 1:**
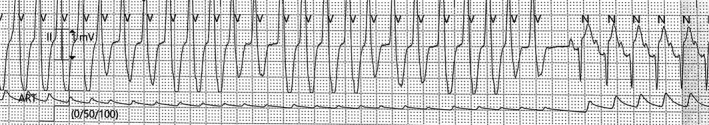
Wide complex tachycardia consistent with idioventricular rhythm associated with loss of pulse on arterial line (ART) tracing

Shortly after the surgery, the patient developed intermittent episodes of accelerated idioventricular rhythm (Figure 1). These episodes were associated with hemodynamic compromise requiring chest compression. The ventricular dysrhythmia worsened in frequency and degree of hemodynamic instability despite escalation in antiarrhythmic therapy. The patient received various combinations of lidocaine, procainamide, amiodarone, and sotalol. Given the inability to medically manage the dysrhythmia, the patient was electively placed on venoarterial extracorporeal membrane oxygenation (ECMO).

**Figure 2:**
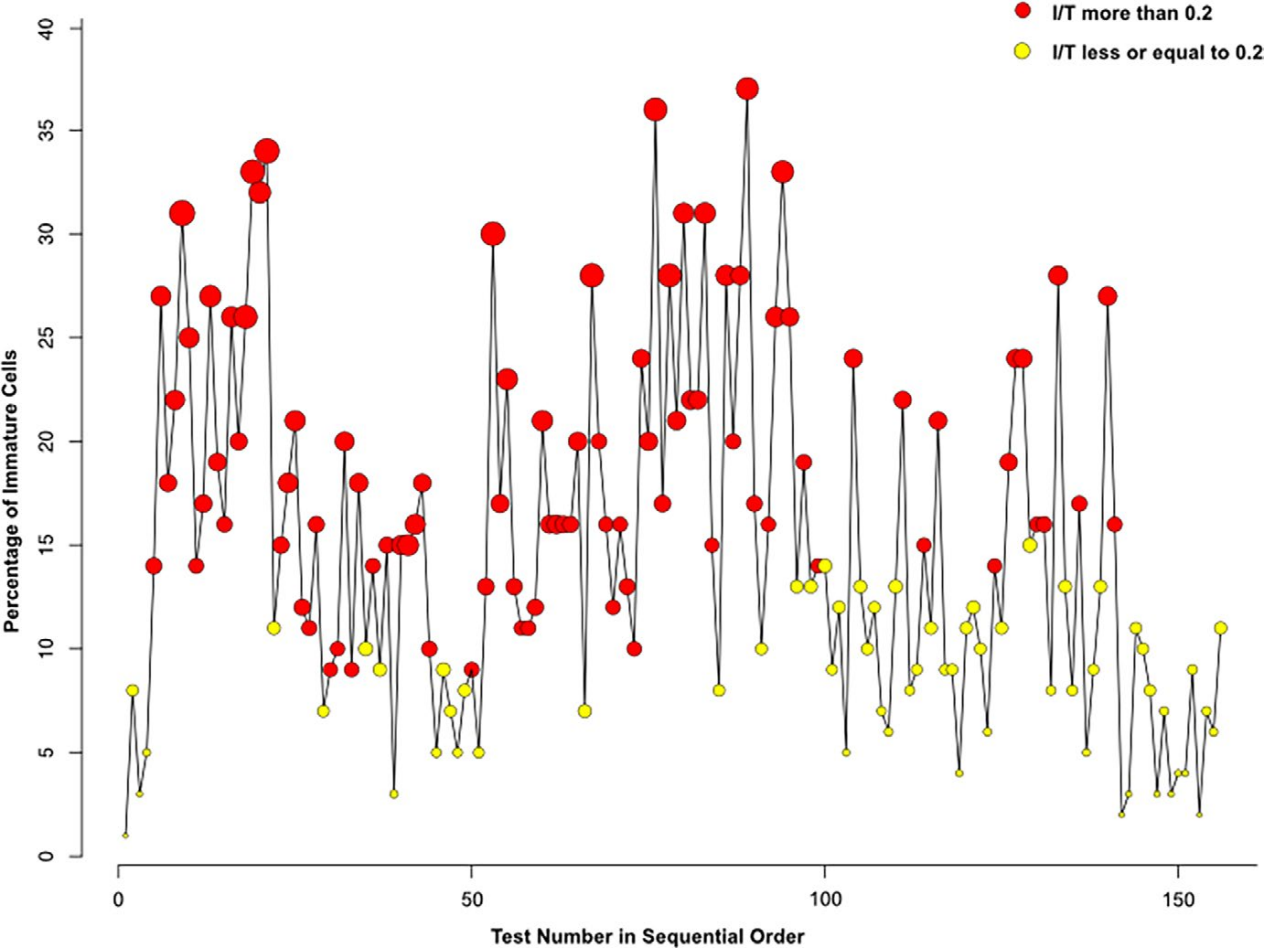
Percentage of immature neutrophils (band neutrophils, blasts, myelocytes, metamyelocytes, and promyelocytes) and ratio of immature to total neutrophils (I/T) on serial complete white blood counts with manual differentiation. The red circles identify the tests where the immature to total neutrophils ratio is greater than 20%. The size of each circle is proportional to the magnitude of the ratio

Following placement on ECMO, the dysrhythmia was completely suppressed with sotalol alone. Unfortunately, his hospital course was complicated by acute kidney injury requiring continuous renal replacement therapy and inability to be weaned off ECMO support due to ECMO‐related complications (volume overload, coagulopathy, acute lung injury associated with blood transfusion). We also noticed persistent monocytosis (12%–41%) and increased number of immature myelocytes on serial complete blood count tests starting from the second week of life (Figure 2, Table 1). These abnormalities persisted despite multiple courses of treatment of presumed systemic infections. The parents decided to withdraw care after 6 weeks of ECMO therapy.

**Table 1:** Weekly percentage of monocytes and ratio of immature to total neutrophils on manual differential blood counts

Weeks of Life	Percentage of Monocytes	Ratio of immature to total neutrophils
2	12%	0.4
3	17%	0.62
4	27%	0.54
5	20%	0.34
6	24%	0.34
7	17%	0.36

### 2.1 Genetic testing

During pregnancy, a cell free DNA study looking from trisomy 13, 18, 21 was normal. After birth, the chromosomal microarray analysis to be certain the baby does not have a chromosomal aneuploidy including deletion of 22q for 7q was normal. Noonan syndrome was suspected given the severe pulmonary stenosis, persistent monocytosis, and “left‐shifted” complete blood counts without any evidence of an infectious process. Postmortem molecular genetic testing performed on previously saved blood specimen revealed that the patient had a heterozygous c.221 C>G (pAla74Gly) mutation in the *RIT1*. This mutation had been described as pathogenic and causative of Noonan syndrome. We do not know whether the parents were tested for this mutation.

## 3 DISCUSSION

Noonan syndrome is part of the RASopathies, a group of clinically overlapping genetic syndromes caused by gain‐of‐function germline mutations of the genes, most of which encode components or regulators of the RAS/mitogen‐activated protein kinase signaling pathway (Roberts et al., 2013). The majority of Noonan syndrome is caused by mutations of the *PTPN11* gene accounting for about 50% of the cases*. RIT1*, a member of the *RAS* subfamily, is the latest gene found to be causative of Noonan syndrome (Aoki et al., 2013; Roberts et al., 2013). To date, there have been reports of 15 different *RIT1* mutations in 141 patients including the patient reported in this article (Aoki et al., 2013; Bertola et al., 2014; Calcagni et al., 2016; Cave et al., 2016; Chen et al., 2014; Cizmarova et al., 2016; Gos et al., 2014; Justino et al., 2015; Koenighofer et al., 2016; Koh et al., 2019; Kouz et al., 2016; Milosavljevic et al., 2016; Nemcikova, Vejvalkova, Fencl, Sukova, & Krepelova, 2016; Ramond et al., 2017; Yaoita et al., 2016). Phenotypically, *RIT1* ***‐***associated Noonan syndrome patients are found to have typical Noonan syndrome facies, milder growth retardation, but higher frequency of cardiac abnormalities when compared to other phenotypes (Calcagni et al., 2016; Cave et al., 2016; Kouz et al., 2016; Yaoita et al., 2016). Only one case of monocytosis has been reported (Nemcikova et al., 2016). More importantly, *RIT1* ***‐***associated Noonan syndrome has more complications and worse prognosis during the perinatal period (Kouz et al., 2016; Yaoita et al., 2016). So far, seven of the identified 141 patients have died.

Our patient did not present with the typical physical characteristics or congenital anomalies commonly described in association with Noonan syndrome other than pulmonary stenosis. On the other hand, he exhibited previously unreported ventricular dysrhythmias. Noonan syndrome was nevertheless suspected based on clinical findings of pulmonary valve stenosis, monocytosis, and increased number of immature white blood cells on peripheral blood smear. Molecular testing detected the heterozygous missense mutation c.221 C>G (pAla74Gly) in *RIT1*. This mutation was first reported by Aoki et al. as pathogenic for Noonan syndrome (Aoki et al., 2013). Including the patient in this report, this mutation has been reported in 18% (25/141) of patients with *RIT1*‐associated Noonan syndrome (Aoki et al., 2013; Bertola et al., 2014; Cave et al., 2016; Cizmarova et al., 2016; Koenighofer et al., 2016; Kouz et al., 2016; Yaoita et al., 2016). This mutation‐specific phenotype has 47% (9/19) prenatal signs and symptoms, 58% (11/19) hypertrophic cardiomyopathy, 75% (15/20) pulmonary stenosis, 50% (10/20) other congenital heart disease, and 11% (2/18) arrhythmia (Aoki et al., 2013; Bertola et al., 2014; Cave et al., 2016; Cizmarova et al., 2016; Koenighofer et al., 2016; Kouz et al., 2016; Yaoita et al., 2016).

### 3.1 Ventricular dysrhythmia in *RIT1*

Atrial dysrhythmias have been increasingly recognized in Noonan syndrome (Levin et al., 2018). On the other hand, lethal ventricular dysrhythmias have only been presumed from cases of unexpected sudden death. These ventricular dysrhythmias have almost always occurred in the setting of hypertrophic cardiomyopathy or pulmonary stenosis (Ramond et al., 2017).

Our patient had unstable accelerated idioventricular rhythm after surgical decompression of the right ventricle and ECMO therapy is unusual since neonatal accelerated idioventricular rhythm is almost universally a benign entity even when associated with congenital heart disease (Freire & Dubrow, 2008).

The underlying etiology of neonatal accelerated idioventricular rhythm remains unclear. Accelerated idioventricular rhythm occurring in the first few days of life might be related to labor‐induced stress, heart immaturity, or disturbance of the autonomic nervous system (Ramond et al., 2017). The presence of an automatic ventricular focus is more likely to be the stimulus for accelerated idioventricular rhythm. Moreover, the attempts of atrial and ventricular burst pacing trials neither stimulated nor terminated the patient's dysrhythmia making reentry a less likely mechanism of his tachycardia. Recent studies have speculated that disruption in cellular calcium homeostasis underlies the automatic atria tachycardia observed in this population (Freire & Dubrow, 2008). However, this patient's dysrhythmia was only responsive to intravenous sotalol, an antiarrhythmic agent which does not alter cellular calcium physiology. The perceived effectiveness of robust potassium channel‐blocking and beta‐blocking properties of sotalol points to an autonomic dysregulation as the possible mechanism of the accelerated idioventricular rhythm associated with this patient with Noonan syndrome. Finally, the possibility of abnormal origin of coronary artery as the etiology of the ventricular dysrhythmia is less likely given no ST segment abnormalities was observed on electrocardiograms and the coronary arteries appeared normal on echocardiograms and left ventricular angiography injection during the catheterization procedure.

### 3.2 Hematologic disorder in *RIT1*

Infants with Noonan syndrome are at risk for several hematologic abnormalities including bleeding secondary to coagulation defects and myeloproliferative disorders mostly associated with *PTPN11* mutations (Roberts et al., 2013; Strullu et al., 2014; Tartaglia et al., 2003). *RIT1* mutations have been anecdotally linked to hematologic disorders such as myeloproliferative process, leukopenia, and neoplasms. One patient had acute lymphoblastic leukemia, one patient had juvenile myelomonocytic leukemia, and one patient had persistent leukopenia and transient monocytosis in the setting of a presumed systemic infection (Aoki et al., 2013; Cave et al., 2016; Nemcikova et al., 2016). Our patient had persistently high percentage of monocytes on serial complete blood counts. More remarkably is the high degree of “left‐shift” on complete blood counts that is persistent and out of character of any infectious process (Figure 2). These abnormalities were observed on and off antibiotic treatments of presumed systemic infections indicating more of a myeloproliferative process in accord with previous phenotypic descriptions.

## 4 CONCLUSION

In summary, we report a case of neonatal Noonan syndrome associated with *RIT1* mutation. The clinical suspicion for Noonan syndrome was based only on the congenital heart defect, persistent monocytosis, and myeloproliferative process as the child lacked all other hallmarks characteristics of Noonan syndrome. However, the patient had an unusually malignant ventricular dysrhythmia that lead to his demise. The case highlights the fact that despite its heterogeneous presentation, *RIT1*‐associated Noonan syndrome can be extremely severe with poor outcome.

## ETHICAL COMPLIANCE

Informed consent was obtained from the parents prior to participation in the research study.

All procedures employed were reviewed and approved by the appropriate institutional review committee. Research data are not shared.

## CONFLICT OF INTEREST

The authors declare no financial or otherwise relevant conflict of interest related to this manuscript.

## AUTHORS CONTRIBUTION

Safwat Aly performed clinical data gathering, literature review, and drafted the manuscript; Kenneth Boyer, Brie‐Ann Muller, and Davide Marini assisted in reviewing and interpretation of clinical data, and approved the final manuscript; Carolyn Jones performed chromosomal microarray and interpretation and assisted in writing of the manuscript; Hoang Nguyen assisted in interpretation of the clinical data and finalized the manuscript.
